# MicroRNA-421 confers paclitaxel resistance by binding to the KEAP1 3′UTR and predicts poor survival in non-small cell lung cancer

**DOI:** 10.1038/s41419-019-2031-1

**Published:** 2019-10-28

**Authors:** Fu-Gang Duan, Mei-Fang Wang, Ya-Bing Cao, Dan Li, Run-Ze Li, Xing-Xing Fan, Imran Khan, Huan-Ling Lai, Yi-Zhong Zhang, Wendy Wen-Luan Hsiao, Xiao-Jun Yao, Qi-Biao Wu, Liang Liu, Yi-Jun Tang, Elaine Lai-Han Leung

**Affiliations:** 1 State Key Laboratory of Quality Research in Chinese Medicine, Macau Institute For Applied Research in Medicine and Health, Macau University of Science and Technology, Macau, SAR China; 2 Department of Respiratory and Critical Care, Taihe Hospital, Hubei University of Medicine, Shiyan, Hubei China; 3 Department of Oncology, Kiang Wu Hospital, Macau, China; 4 Department of Pathology, Taihe Hospital, Hubei University of Medicine, Shiyan, Hubei China; 5 Zhuhai Hospital of Integrated Traditional Chinese and Western Medicine, Zhuhai, China

**Keywords:** Non-small-cell lung cancer, Mechanisms of disease

## Abstract

MicroRNAs regulate post-transcriptional gene expression and play important roles in multiple cellular processes. In this study, we found that miR-421 suppresses kelch-like ECH-associated protein 1(KEAP1) expression by targeting its 3′-untranslated region (3′UTR). A Q-PCR assay demonstrated that miR-421 is overexpressed in non-small cell lung cancer (NSCLC), especially in A549 cells. Consistently, the level of miR-421 was higher in clinical blood samples from lung cancer patients than in those from normal healthy donors, suggesting that miR-421 is an important lung cancer biomarker. Interestingly, overexpression of miR-421 reduced the level of KEAP1 expression, which further promoted lung cancer cell migration and invasion, as well as inhibited cell apoptosis both in vivo and in vitro. Furthermore, knockdown of miR-421 expression with an antisense morpholino oligonucleotide (AMO) increased ROS levels and treatment sensitivity to paclitaxel in vitro and in vivo, indicating that high miR-421 expression may at least partly account for paclitaxel tolerance in lung cancer patients. To find the upstream regulator of miR-421, one of the candidates, β-catenin, was knocked out via the CRISPR/Cas9 method in A549 cells. Our data showed that inhibiting β-catenin reduced miR-421 levels in A549 cells. In addition, β-catenin upregulation enhanced miR-421 expression, indicating that β-catenin regulates the expression of miR-421 in lung cancer. Taken together, our findings reveal the critical role of miR-421 in paclitaxel drug resistance and its upstream and downstream regulatory mechanisms. Therefore, miR-421 may serve as a potential molecular therapeutic target in lung cancer, and AMOs may be a potential treatment strategy.

## Introduction

Lung cancer is the most common cause of cancer death worldwide^1,2^. Non-small cell lung cancer (NSCLC) is the most common type, accounting for ~85% of all lung cancers^3^. Surgery, radiotherapy, chemotherapy, targeted therapy and immunotherapy are common current therapies for lung cancer. Chemotherapy treatment for NSCLC includes cisplatin, carboplatin (Paraplatin), paclitaxel (Taxol), docetaxel (Taxotere), gemcitabine (Gemzar), and pemetrexed (Alimta)^4–6^. Among these, paclitaxel has high anti-tumour activity and is often used alone or in combination as an important chemotherapeutic agent for the treatment of NSCLC^7,8^. One of the accepted anti-tumour mechanisms of paclitaxel is that it binds to the β-subunit of tubulin to prevent the formation of microtubules, thereby affecting the cell cycle and eventually promoting cell death^9,10^. Another mechanism of paclitaxel is involved in regulating the Akt pathway^11^, the MAPK pathway^12^, Bcl-2^13^, and caspase-3^14^. Unfortunately, patients commonly develop acquired paclitaxel resistance, which has become a critical clinical problem for the successful treatment of lung cancer. However, the mechanism of paclitaxel resistance is still unclear. Reactive oxygen species (ROS) are collectively referred to as chemical substances that contain oxygen and participate in biological processes. Several studies have shown that different ROS levels can induce different biological processes^15^. It is generally believed that low ROS levels promote tumour proliferation, survival and metastasis^16–18^. However, more than a certain amount of ROS can promote tumour apoptosis^19,20^. Increasing evidence suggests that ROS play an essential role in regulating target-therapy resistance^21–23^, and targeting the redox regulatory pathway has become a therapeutic strategy. In addition, ROS are associated with chemical resistance^24,25^. KEAP1 (kelch-like ECH-associated protein 1) is involved in the regulation of ROS. KEAP1 is a substrate adaptor protein that binds substrates via the CuI3-containing E3 ubiquitin ligase complex and degrades substrates via the proteasome pathway^26^. KEAP1 interacts with the transcription factor nuclear factor (erythroid-derived 2)-like 2 (Nrf2)^27^. Under oxidative stress conditions such as ROS stimulation, KEAP1 modification releases Nrf2 from the KEAP1–Cul3 E3 ligase complex^28^. Nrf2 then translocates into the nucleus and cooperates with a small-Maf binding partner to activate a DNA promoter, the antioxidant response element (ARE)^29,30^. In addition, KEAP1 is associated with drug resistance. Loss of KEAP1 function leads to chemical resistance in prostate cancer^31^. Inhibition of Nrf2 expression by overexpressing KEAP1 sensitized both cell culture and mouse models to chemotherapeutic treatments^32^. USP15 negatively regulates Nrf2 through deubiquitination of KEAP1 and affects paclitaxel resistance^33^. Notably, KEAP1 is a well-known antioxidant gene that plays an important regulatory role in cancer survival or response to drug resistance.

MicroRNA (miRNA) is a type of non-coding RNA that modulates protein synthesis and post-transcriptional expression levels^34^. MiRNAs bind to part of the 3′UTR of targeted mRNAs. The matching of miRNAs to target sequences leads to transcriptional repression^35^. There are multiple studies on the function of miRNAs in the regulation of cancer via various mechanisms^36^. MiR-421 has been found to regulate cell proliferation, invasion, migration, cell cycle progression, colony formation, apoptosis, and radiotherapy resistance in cancer^37–40^. In breast cancer, miR-421 promotes proliferation by targeting PDCD4^41^. MiR-421 also promotes the development of breast cancer by targeting caspase-10^42^. MiR-421 promotes the proliferation and invasion of gastric cancer by inhibiting the expression of CLDN11^43^. According to different techniques, the serum miR-421 levels are higher in GC patients than in normal subjects^44^. Similarly, clinical studies have shown that high expression of miR-421 is associated with poor prognosis in patients with non-small cell lung cancer^45^. Furthermore, miR-421 promotes the development of lung cancer. In recent years, few studies have reported the mechanism by which miRNAs target KEAP1 to regulate tumourigenesis. Some studies have reported that miR-23a protects itself from ROS in leukaemic cells by targeting the KEAP1 3′UTR^46^. However, little is known about the role of miR-421 in NSCLC and whether miRNA affects drug resistance through regulating KEAP1.

It is well known that Wnt/β-catenin regulates cancer progression and metastasis in different tumours^47^. The Wnt signalling pathway activates the transcriptional promoter region of miRNA through β-catenin binding, thereby increasing miRNA expression. miR-30e is activated by β-catenin/TCF4 complex transcriptional activation during intestinal cell differentiation in rats^48^. Similarly, β-catenin/TCF4 promotes the expression of miR-21 in a STAT3-dependent manner, thereby promoting cell invasion in glioma^49^. Wnt/β-catenin signalling upregulates miR-770 to increase cell proliferation in HCC^50^. Wnt/β-catenin signalling increases miR-183/96/182 expression levels to enhance cell invasion^51^. However, to the best of our knowledge, the role and regulatory mechanism through which Wnt/β-catenin is mediated by miRNAs and their functional effects remain unclear in NSCLC.

In this study, we explored the role of miR-421 and its correlation with paclitaxel drug resistance in NSCLC and its related up/downstream pathways.

## Materials and methods

### Cell lines and reagents

The human normal lung epithelial and fibroblast cell lines BEAS-2B and CCD19-lu and the human NSCLC cell lines A549, H358, H1650, H460, and H1975 were purchased from the American Type Culture Collection (Manassas, VA, USA). Human normal lung fibroblast BEAS-2B cells were grown in BEGM, whereas the NSCLC cell lines were all grown in RPMI 1640 medium (SGC-7901) from Gibco (Carlsbad, CA, USA). All culture media contained 10% FBS with 100 μg/ml streptomycin and 100 U/ml penicillin, and the cells were cultured at 37 °C in a humidified atmosphere containing 5% CO_2_. Paclitaxel was purchased from Mu Biotechnology Co. Ltd. (Guangzhou, GD, China).

### Cytotoxicity assay

Approximately 3000 cells were cultured in 96-well plates overnight for cell adhesion and treated with DMSO or different concentrations of paclitaxel for 48 h. Then, 10 μl of MTT (5 mg/ml, Sigma) was added to each well for 4 h at 37 °C; then, the reagent was removed, and the crystals were dissolved in 100 μl of a resolving solution (DMSO). The absorbance at 570 nm was measured using a microplate reader from Tecan (Morrisville, NC, USA). Cell viability was calculated relative to that of the untreated control cells. At least three independent experiments were performed.

### RNA isolation and real-time PCR

Total RNA was extracted from cultured cells using a mirVana miRNA isolation kit purchased from Invitrogen (Carlsbad, CA, USA). All cDNA was synthesized using M-MLV reverse transcriptase (Invitrogen) according to the manufacturer's protocol. The expression of miR-421 in cell lines was determined by quantitative real-time PCR (qRT-PCR). U6 expression was used as an internal control. All primers were synthesized by Sangon Biotech (Shanghai, SH, China) Co., Ltd. AMO-421 and control AMO were purchased from Invitrogen (Carlsbad, CA, USA). All primers are shown in Table 1. Serum RNA extraction was performed using the miRcute Serum/Plasma miRNA Isolation Kit from Tiangen (Beijing, BJ, China).

**Table 1:** Primer list

	Forward	Reverse	Restriction sites
U6-RT	GTCGTATCCAGTGCAGGGTCCGAGGTGCACTGGATACGACAAAATATGGAAC		−
miR-421-RT	GTCGTATCCAGTGCAGGGTCCGAGGTGCACTGGATACGACGCGCCCA		−
U6	TGCGGGTGCTCGCTTCGGCAGC	CCAGTGCAGGGTCCGAGGT	−
MiR-421	TGCGGATCAACAGACATTAATTGGGC	CCAGTGCAGGGTCCGAGGT	−
KEAP1	AGCGCTACGATGTGGAAACA	GTCCAGGAACGTGTGACCAT	−
GAPDH	AAGGTGAAGGTCGGAGTCA	AATGAAGGGGTCATTGATGG	−
KEAP1 3′UTR	ACGTACGCTAGCGAAGCAGATTGACCAGCAGA	ACGTACCTCGAGAGTTAGTCTGTTCTTTATTTTCC	NheI/Xhol
KEAP1 Mutaion 3UTR	AAATAACTGTCCATCCGGTG	TAAGTAACCCTGTAATTTTCCAAGG	−
miR-421 promoter	ACGCTAGCTAGCGGCCCTGAGCAGTGCAGCAG	ACGCCGCTCGAGAAACATTTAATGAGGCCTAC	NheI/Xhol
g-β-catenin A	CACCGACCACAGCTCCTTCTCTGAG	AAACCTCAGAGAAGGAGCTGTGGTC	BbsI
g-β-catenin B	CACCGCAGAATGGATTCCAGAGTCC	AAACGGACTCTGGAATCCATTCTGC	BbsI

### Plasmid construction

The full-length KEAP1 3′UTR was amplified by PCR and cloned into the pmirGLO dual-luciferase miRNA target expression vector with an endonuclease (NheI/Xhol). KEAP1 3′UTR deletion vector construction was performed using the NEB Q5 Site-Directed Mutagenesis Kit (Ipswich, MA, USA). 1 kb of the gene upstream of the KEAP1 gene start codon was cloned into the pGL3 luciferase reporter vector with an endonuclease (NheI/Xhol). All constructed vectors were confirmed by DNA sequencing. All primers are shown in Table 1.

### Western blotting analysis

Cells were lysed in RIPA buffer (50 mmol/L Tris–HCl, 150 mmol/L NaCl, pH 8.0, 0.1% SDS, 1% Triton X-100, and 1% deoxycholate) with a complete protease inhibitor from Roche (Basel, UK) for 10 min on ice. Concentrations of the total protein extracts were measured using a Bio-Rad DCTM protein assay kit. Then, the protein samples (30 µg) were separated by SDS–PAGE and transferred to nitrocellulose (NC) membranes. The membranes were incubated with various primary antibodies, including phospho-AKT (CST), phospho-ERK (CST), and KEAP1 (Abcam) at a 1:1000 dilution and GAPDH (Santa Cruz) at a 1:2000 dilution, overnight at 4 °C. After washing the membranes 3 times (5 min/time) with TBST buffer, the appropriate anti-rabbit or anti-mouse secondary antibodies (CST) were incubated with the membranes at 1:10,000 dilution at room temperature for 1 h. GAPDH was used as a loading control.

### Lentiviral production and infection

The miR-421-overexpressing lentivirus plasmid was supplied by GenePharma (Shanghai, SH, China). The lentiviral vector and its packaging vector were co-transfected into HEK293T cells for packaging by Lipofectamine 2000. Briefly, 1 × 10^6^ HEK293T cells were transfected with 5 µg of miR-421 expressing plasmid together with 1 µg of pTAT pVSV-G pREV pGAG (the packaging vector). After 4 h of incubation, the transfection medium was replaced with fresh culture medium. After 48 h, the supernatant was harvested by centrifugation at 1500 rpm for 5 min. The supernatant was filtered through a membrane with a 0.45-µm filter. A549 cells were infected with the miR-421-overexpressing lentivirus or the corresponding mock lentivirus. The packaged virus and 8 μg/mL Polybrene® (Sigma-Aldrich, St Louis, MI, USA) were added to the cells for 4–8 h and then replaced with fresh medium. After 48 h, the infected cells were subjected to selection with 2 μg/mL puromycin.

### CRISPR-Cas9 sgRNA design and sgRNA cloning

The two targeting sequences used in this study were GGACTCTGGAATCCATTCTG and ACCACAGCTCCTTCTCTGAG. DNA oligos were synthesized and cloned into pX335-U6-Chimeric_BB-CBh-hSpCas9n (D10A) (Addgene 42335). A549 cells were transfected with the constructs using Lipofectamine 2000 according to the manufacturer’s protocol. All DNA oligos are shown in Table 1.

### Migration, invasion and wound healing assays

In brief, migration and invasion were detected using 24-well Millicell Hanging Cell Culture Inserts (Corning, St Louis, MI, USA) with a pore size of 8.0 µm. The inset was coated with Matrigel (BD Biosciences, San Jose, CA, USA) for the invasion assays according to the manufacturer's protocol. Cells were cultured at 37 °C for 24 h for the migration assays and for 24 h for the invasion assays. The cells on the upper membrane were removed, and the cells on the bottom of the microporous membrane were stained with crystal violet. For the wound healing assays, cells were cultured in 6-well plates and scratched with 10-µl plastic pipette tips. The cell debris was washed away with PBS, and the migration distance was measured using a microscope at 24 h.

### Detection of ROS production

ROS generation was detected with a DCFCA fluorescence probe using a flow cytometer. Briefly, A549 cells (1 × 10^5^ cells/well) were seeded in a 6-well plate and transfected with control AMO or AMO-421 according to the transfection protocol. After 24 h, the transfected cells were separated by trypsin and washed twice with PBS. The cells were incubated with 10 μM DCFCA for 30 min at 37 °C in the dark, and the levels of the fluorescently stained cells were then analysed using a FACS BD Aria III flow cytometer (BD Biosciences, San Jose, CA, USA).

### Cell apoptosis assays

Apoptosis was measured using an annexin V-FITC apoptosis detection kit (BD Biosciences, San Jose, CA, USA). Briefly, A549 cells (1 × 10^5^ cells/well) were cultured in a 6-well plate and transfected with control AMO or AMO-421 according to the transfection protocol. Apoptosis was detected after the addition of paclitaxel. Cells were trypsinized, washed 3 times with PBS, stained with 100 µl of binding buffer containing 2 µl of annexin V FITC (2.5 µg/ml) and 5 µl of propidine iodide (50 µg/ml) and incubated in the dark at room temperature for 15 min. The stained cells were measured quantitatively using a BD Aria III flow cytometer (BD Biosciences, San Jose, CA, USA). The data were analysed by Flow Jo software.

### Mouse xenograft assays

All animal experiments were performed in accordance with institutional animal care guidelines and protocols approved by the committee. Approximately 2 × 10^6^ A549 cells were resuspended in PBS and mixed with Matrigel in a 2:1 ratio, and 150 µl of the mixture was injected subcutaneously into the right flanks of 6-week-old nude mice. Xenografts were grown to ~60 mm^3^, and 5 mice per group were treated with vehicle (2% DMSO, 40% PEG400, and 5% Tween 80 in normal saline) or paclitaxel (20 mg/kg) via intraperitoneal (ip) injection daily for 21 days. AMO-control and AMO-421 intratumoural injections were performed once a week^52^. Tumour volumes were measured every 3 days.

### Clinical sample collection and immunohistochemistry

NSCLC tissue specimens from 2015 to 2017 were used. These patients underwent tumour resection at Taihe Hospital. The tumour tissues were fixed in 4% formalin and embedded into tissue blocks. Serum samples were also collected from NSCLC advanced patients and healthy donors. The serum was stored in a −80 °C freezer. Immunohistochemistry analyses were performed using a DAKO EnVisionTM system (Dako, Glostrup, Denmark). Experimental steps were carried out according to the appropriate instructions. The following antibodies were used: KEAP1 from Abcam (Cambridge, MA, USA). Semi-quantitative analysis was used for the tissue staining results. The intensity was scored as follows: 0 negative; 1 weak; 2 moderate; and 3 strong. According to the degree of staining, negative and weak were considered low expression, and moderate and strong were considered high expression.

### Luciferase reporter assays

HEK293 cells were cotransfected with the indicated reporter vectors and miR-421 (50 nM) or miR-NC (50 nM). Luciferase activity was measured with the Dual-Glo assay (Promega, Madison, WI, USA). The results are expressed as a normalized ratio of *Firefly* to *Renilla* luciferase activity. The data are presented as the mean ± SD from at least 3 samples per data point.

### Statistics

The data are expressed as the mean ± SD of three individual experiments. Differences between groups were established by one-way analysis of variance (ANOVA) followed by Bonferroni’s test to compare all pairs of columns. The results were deemed to be significant at *P* < 0.05. The relationship between KEAP1 expression and clinicopathological features was analysed by chi-square test using SPSS (Chicago, IL, USA).

## Results

### MiR-421 suppresses KEAP1 expression by targeting the 3′UTR of KEAP1

**Fig. 1:**
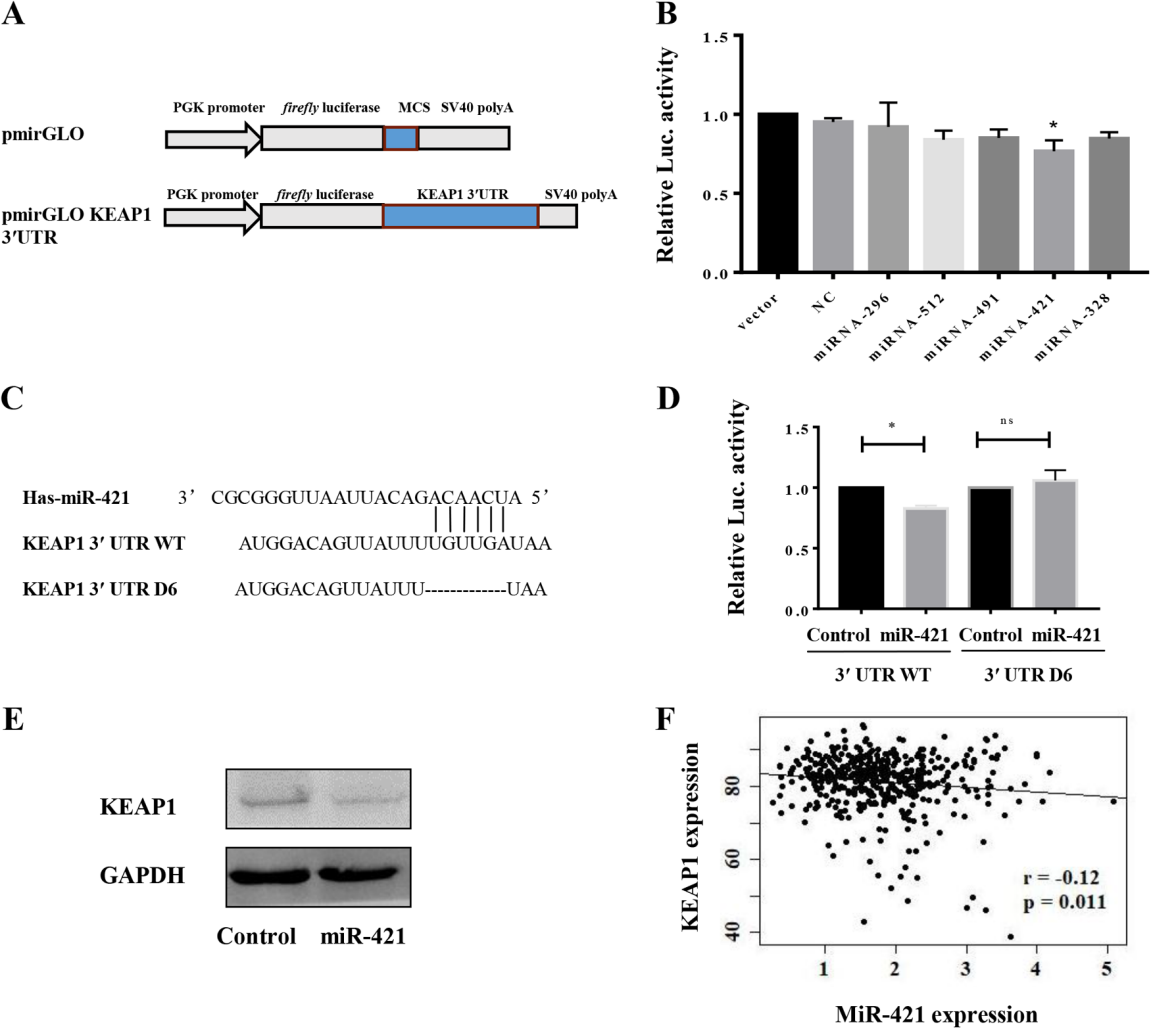
Identification of KEAP1 as a miR-421 target gene. **a** Luciferase constructs containing the KEAP1 3′UTR. **b** Screening of miRNAs targeting KEAP1 by luciferase reporter assays. **c** Models of luciferase constructs containing the WT 3′UTR of KEAP1 and del6 (D6) 3′UTR of KEAP1. **d** HEK293 cells were co-transfected with NC and miR-421 with the WT or Mut 3′UTR. Luciferase activity was assayed 24 h after transient co-transfection. *Renilla* luciferase activity was measured and normalized to *firefly* luciferase activity. *P < 0.05 vs miR-421. **e** The protein level of KEAP1 in A549 cells was analysed by western blotting after transfection with miR-421. **f** LUADs from The Cancer Genome Atlas (TCGA). Expression values were retrieved from an RNAseq dataset (Illumina HiSeq)

To explore the possibility that microRNAs might regulate KEAP1 expression, TargetScan (http://www.targetscan.org) and other online databases were used to predict a panel of possible miRNAs that can regulate KEAP1. Bioinformatics analysis suggested that KEAP1 might be a target for the following microRNAs: miR-296-3p, miR-512-3p, miR-491-5p, miR-421, and miR-328-3p. To further determine whether KEAP1 was a direct downstream target of these microRNAs, the 3′UTR of KEAP1 was cloned downstream into the pmirGLO vector for dual-luciferase miRNA target screening (Fig. 1a). Next, we performed a luciferase reporter assay following co-transfection of a reporter vector with mimic miRNA into HEK293 cells. A significant reduction in luciferase activity was observed in the presence of miR-421, while the luciferase activity of the negative control and the other miRNAs was not significantly altered (Fig. 1b). In addition, vectors with deletion of 6 nucleotides of the seed sequence (D6) binding sites of KEAP1 were also constructed (Fig. 1c). Deletion of 6 nucleotides of the targeting sequence (D6) resulted in restoration of miR-421-mediated luciferase activity (Fig. 1d), suggesting the presence of a direct interaction between the six nucleotides of the KEAP1 3′UTR and miRNA-421. To further confirm that KEAP1 is a target of miR-421, we detected the endogenous KEAP1 protein level by immunoblot after transiently transfecting mimic miR-421 into A549 cells. As shown in Fig. 1e, the expression level of KEAP1 decreased after miR-421 overexpression. Similarly, Pearson’s correlation analysis of miR-421 and KEAP1 expression from the TCGA database showed that there is a negative correlation between the two genes (Fig. 1f). Together, these results strongly indicated that miR-421 negatively regulates KEAP1.

### Increased miRNA-421 expression in patient plasma samples and low KEAP1 expression are associated with worse outcomes in lung cancer

**Fig. 2:**
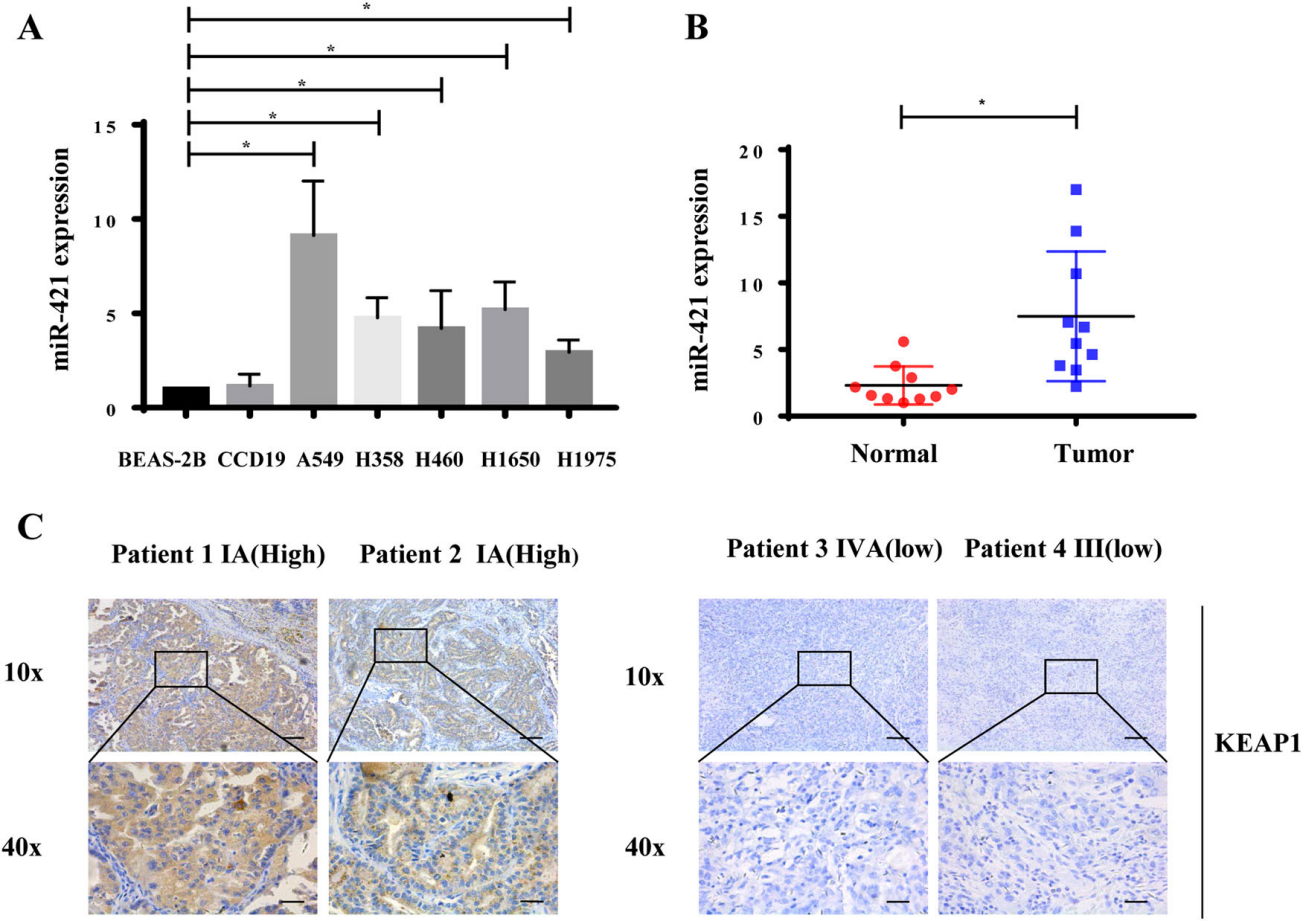
MiR-421 is increased in lung cancer. **a** Q-PCR showed that expression of miR-421 was higher in lung cancer cell lines (A549, H1975, H1650, H460, H358) than in the human lung epithelial cell line BEAS-2B. The columns indicate independent experiments. **b** Scatter dot plots showing that the expression of miR-421 was significantly higher in lung cancer tumour serum samples than in non-tumour serum samples. *P < 0.05. *N* = 10 for each group. **c** Immunohistochemistry analysis of KEAP1 expression in lung cancer tissues with different clinical stages

To investigate the potential role of miR-421 in lung cancer, we examined the expression of miR-421 in a panel of lung cancer cell lines and a pair of normal lung cells. The results showed that the expression of miR-421 was significantly higher in lung cancer cells than in normal cells, while A549 cells showed the highest expression levels, so we used A549 cells for the experimental model for the rest of the studies (Fig. 2a). Next, we determined the clinical significance of miR-421 expression in serum samples from lung cancer patients and healthy controls. As shown in Fig. 2b, the expression of miR-421 was substantially increased in lung cancer patient serum samples. Given the critical role of KEAP1 in several other cancer types, we decided to investigate the clinical relevance of KEAP1 expression in clinical lung cancer tumours, and we detected the protein level of KEAP1 in lung cancer patient samples by the immunohistochemical method. Notably, the protein level of KEAP1 was higher in patients with early stage (I) lung cancer than in patients with late-stage (III and IV) tumours (Fig. 2c, Table 2). The lower the level of KEAP1 was, the worse the stage was. These results indicated that miR-421 downregulation of KEAP1 expression is a critical event during tumour progression.

**Table 2:** Relationship between expression of KEAP1 and clinicopathological parameters in 129 patients with stage I–IV lung cancer

Features	All cases	KEAP 1 expression		F/X^2^ *p-value*
		Low	High	
Total	129 (100.0%)	68 (52.7%)	61 (47.3%)	
Gender				1.41 (*p* = 0.17)
Male	103 (100.0%)	57 (55.3%)	46 (44.7%)	
Female	26 (100.0%)	11 (42.3%)	15 (57.7%)	
Ages				0.02 (*p* = 0.54)
≤ 65	103	54	49	
> 65	26	14	12	
Smoking history				3.05 (*p* = 0.06)
Yes	88 (100.0%)	51 (58.0%)	37 (42.0%)	
No	41(100.0%)	17 (41.5%)	24 (58.5%)	
Pathological patterns				3.8 (*p* = 0.038)
Adenocarcinoma	73 (100.0%)	33 (45.2%)	40 (54.8%)	
Squamous carcinoma	56 (100.0%)	35 (62.5%)	21 (37.5%)	
Cell differentiation				**4.62 (*p*** **=** **0.10)**
Poorly	27 (100.0%)	18 (66.7%)	9 (33.3%)	
Moderately	92 (100.0%)	43 (46.7%)	49 (53.3%)	
Well	10 (100.0%)	7 (70.0%)	3 (30.0%)	
Tumor stage				**14.82 (*p*** **=** **0.002)**
I	62 (100.0%)	22 (35.5%)	40 (64.5%)	
II	36 (100.0%)	25 (69.4%)	11 (30.6%)	
III	27 (100.0%)	19 (70.4%)	8 (29.6%)	
IV	4 (100.0%)	2 (50.0%)	2 (50.0%)	

### MiR-421 plays an oncogenic role in lung cancer

**Fig. 3:**
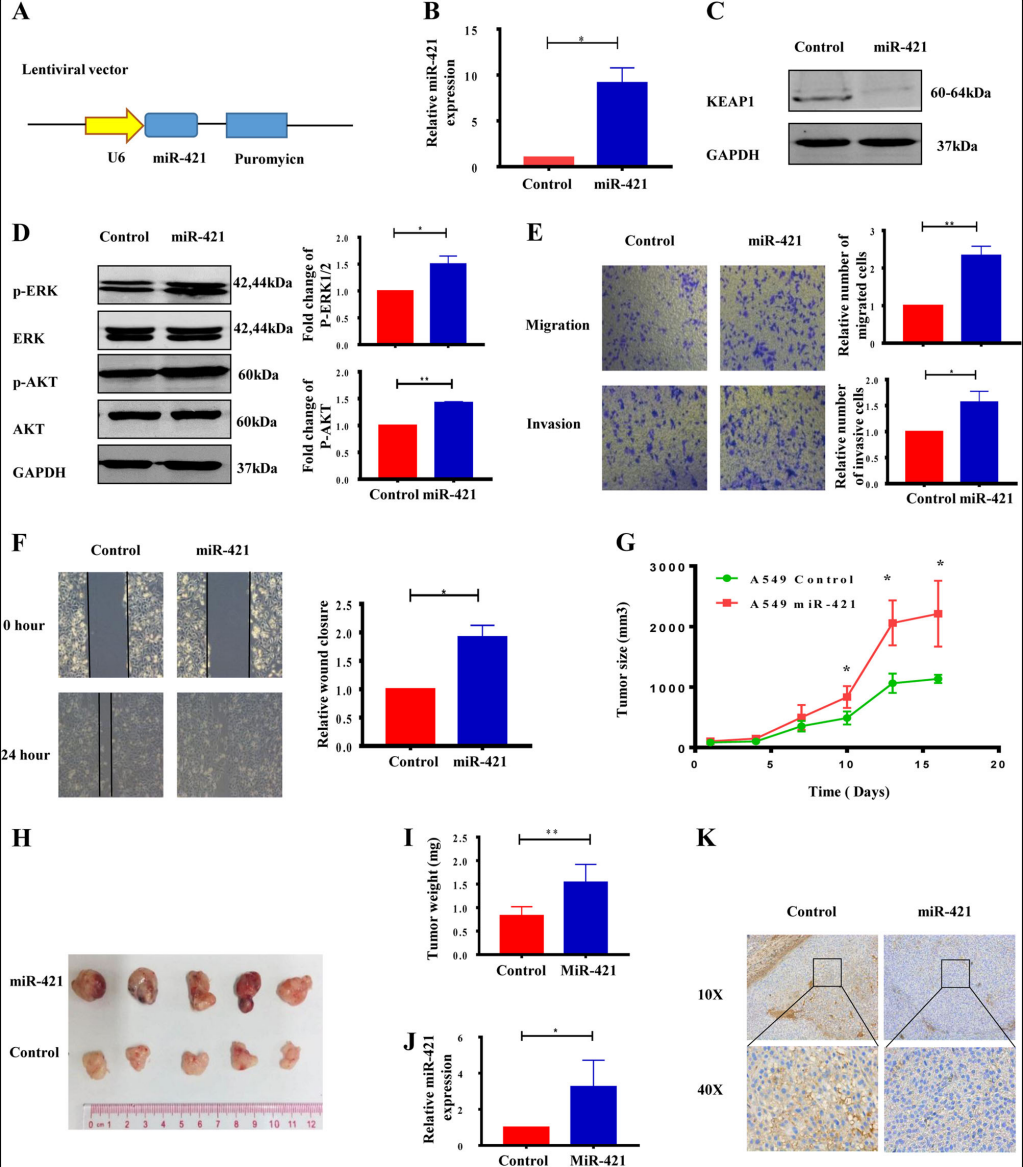
MiR-421 plays an oncogenic role in lung cancer. **a** Schematic diagram of lentiviral vector construction. **b** Detection of miR-421 expression in stable cells by real-time PCR. **c** Western blot analysis of KEAP1 in a cell line overexpressing miR-421. **d** miR-421 increases the levels of p-AKT and p-ERK. Cell extracts were prepared and analysed by western blotting with antibodies against p-AKT and p-AKT. GAPDH was used as a loading control. **e** Migration and invasion assay were preformed in control and mir-421 overexpression stable cells. **f** Wound healing assay using miR-421 overexpression stable cells and control cells. **g** Tumour growth curve showing stable expression in A549 cells infected with WT A549 and miR-421 for 21 days. *P < 0.05 vs. the control group. The data represent the mean ± SD (*n* = 5). **h** Tumour sizes are shown. **i** Tumour weights were significantly higher in the miR-421 groups than in the control groups. **j** The relative gene expression of miR-421 was confirmed by Q-PCR analysis in the miR-421 groups and in the control groups. **k** Immunohistochemical analysis of KEAP1 expression in the control group and miR-421 overexpression group

To further confirm the function of miR-421 in lung cancer, a stable cell line overexpressing miR-421 was constructed using A549 cells and lentivirus transfection (Fig. 3a). The expression level of miR-421 was significantly enhanced in the stable line (Fig. 3b); consistently, KEAP1 was decreased dramatically (Fig. 3c). Given the critical role of the AKT/ERK pathway in lung cancer, we examined the effect of miR-421 on signalling pathways. As shown in Fig. 3d, the levels of p-AKT and p-ERK were higher in the miR-421 overexpression groups than in the negative control groups, suggesting that miR-421 promotes the activation of the AKT anti-apoptotic and ERK cell proliferation signalling pathways. To determine if miR-421 is critical for cell migration and invasion, we performed transwell assays and wound healing assays. As shown in Fig. 3e, miR-421 overexpression cells showed faster migration and invasion ability than control cells according to transwell assays. Similarly, the miR-421 overexpression group significantly increased the wound healing ability (Fig. 3f). We also have added two more lung cancer cell lines (H1975 and H358) to support our conclusion. The level of p-AKT and p-ERK increased in miR-421 overexpression groups compared to negative control groups in H1975 and H358 cell lines as in A549 (Supplementary Fig. S1A). In addition, as shown in Supplementary Fig. S1B, miR-421 overexpression cells showed faster migration than that of control cells by transwell assays in H1975 and H358, which is consistent with that in A549. Similarly, miR-421 overexpression group significantly increased the ability in wound healing in H1975 and H358 (Supplementary Fig. S1C). These data demonstrated that miR-421 promotes lung cancer cell migration and invasion in vitro.

We next examined the effects of miR-421 on the tumour formation potential of NSCLC. MiR-421-overexpressing cells and wild-type A549 cancer cells were injected subcutaneously into nude mice. In vivo data revealed that both tumour size and tumour weight were enhanced in the miR-421 overexpression group (Fig. 3g, h, i). Real-time PCR revealed a significant increase in the expression of mature miR-421 in the tumours from mice injected with miR-421 overexpression cells compared with that in the tumours from mice injected with control cells (Fig. 3j). Furthermore, immunohistochemical analysis demonstrated that overexpression of miRNA-421 reduced KEAP1 expression in tumours (Fig. 3k). All of these results proved that miR-421 promotes the proliferation, invasion and migration of lung cancer.

### MiRNA-421 reduces paclitaxel sensitivity

**Fig. 4:**
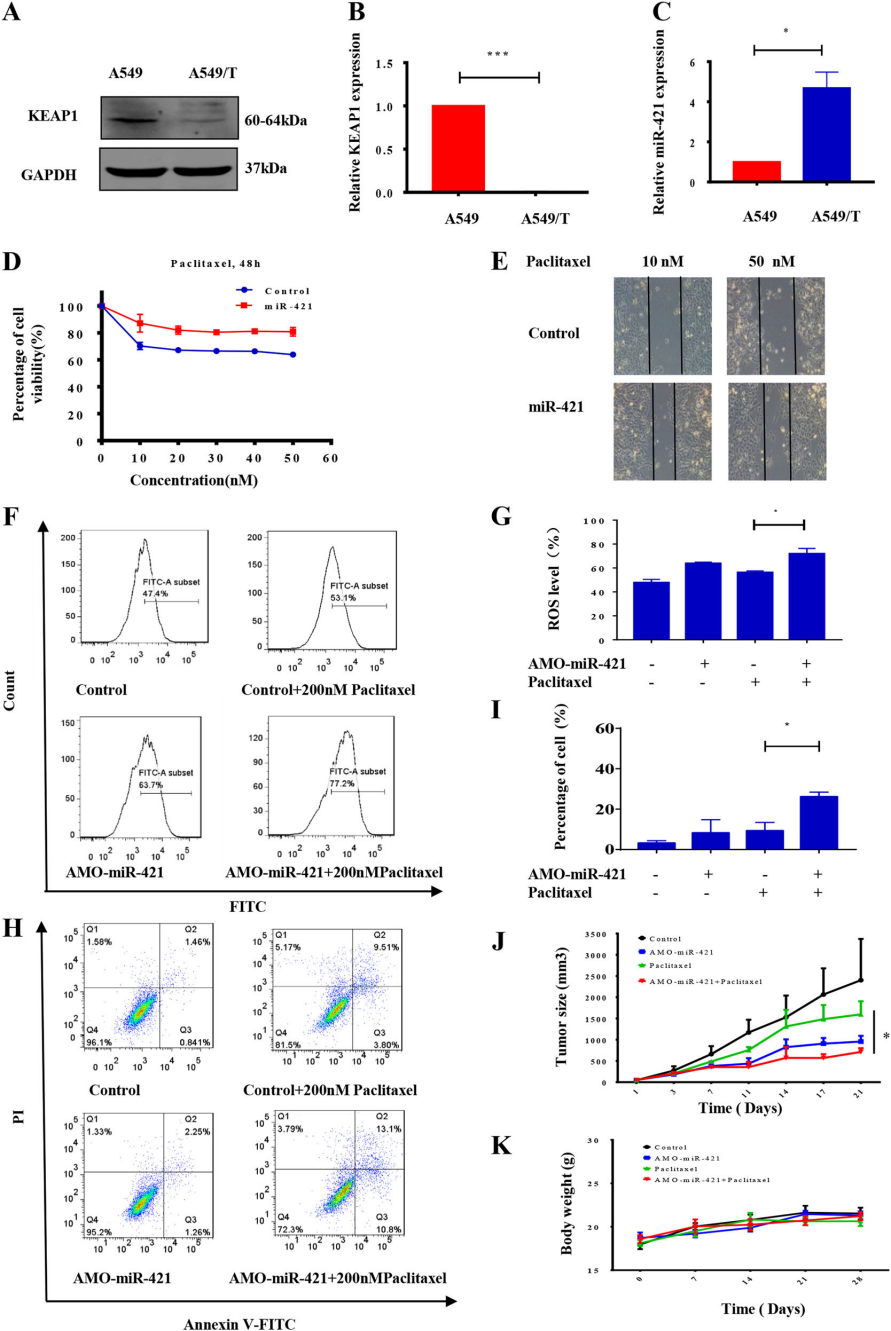
MiR-421-KEAP1 mediates drug resistance to paclitaxel in lung cancer. **a** WB analysis of KEAP1 expression in A549 and A549T cells. **b** Detection of KEAP1 mRNA levels in A549 and A549T cells via real-time PCR. **c** Examination of endogenous miR-421 levels by real-time PCR in A549 and A549T cells. **d** Cell viability was measured in A549 and miR-421 overexpression stable A549 cells after treatment with paclitaxel for 24 h by MTT assay. **e** Wound healing assays were performed in WT and miR-421 overexpression stable A549 cells after treatment with 10 nM or 50 nM paclitaxel. **f**, **g** After transfection with AMO-miR-421 for 24 h, ROS generation was detected by flow cytometry after DCFDA staining. The results are expressed as the mean ± SD of 3 independent experiments, **P *<* 0.01 when compared with control. **h**, **i** A549T cells or cells with AMO-421 were treated with vehicle or 200 nM paclitaxel for 24 h, and cell apoptosis was measured by Annexin V/PI double staining and flow cytometry. The data are presented as the mean ± SD. *P < 0.05. **j** After injection with A549 cells, mice were treated with vehicle, paclitaxel, AMO-miR-421 or paclitaxel combined with AMO-miR-421. A tumour growth curve is shown for 28 days. The data represent the mean ± SD (*n* = 5). **k** Quantification of the body weights of mice from each group. The data are presented as the mean ± SD; *n* = 5 per group

Some studies have reported that KEAP1 is associated with chemical resistance^32,33^. To determine whether miR-421 and KEAP1 contribute to paclitaxel sensitivity in NSCLC, we assessed the levels of KEAP1 and miR-421 in wild-type A549 cells and their counterpart paclitaxel-resistant cells (A549T). As shown in Fig. 4a, b, both the protein and mRNA levels of KEAP1 were significantly lower in paclitaxel-resistant cells than in wild-type A549 cells, suggesting that KEAP1 loss was related to paclitaxel resistance. In contrast, the expression level of miR-421 was higher in A549T cells than in wild A549 cells (Fig. 4c). To understand the potential role of miR-421 in the regulation of paclitaxel resistance in lung cancer, we transfected miR-421 into A549 cells. The cytotoxicity of paclitaxel in wild type A549 and miR-421-transfected A549 cells was determined by MTT assays. As expected, a reduction in the percentage of apoptotic cells was observed in the miR-421-overexpressing A549 cells, indicating that miR-421 overexpression rescues paclitaxel cytotoxicity (Fig. 4d). Similarly, cells with miR-421 overexpression showed stronger migration ability after treatment with paclitaxel according to the wound healing assay results (Fig. 4e). To further examine the effect of miR-421 on paclitaxel resistance, we used synthetic anti-microRNA oligonucleotides (AMOs) to knock down miR-421. AMOs inhibit miRNA function through high-affinity binding with target miRNA^53^. Thus, AMOs are a primary tool used to study miRNA biological functions in vitro and in vivo^54,55^. Because KEAP1 is related to antioxidation, we assumed that knockdown of miR-421 would affect intracellular ROS levels. As expected, the results showed that knocking down miR-421 with AMO increased ROS levels (Fig. 4f, g). Given that overexpression of miR-421 promotes cell survival after treatment with paclitaxel, we hypothesized that a miR-421 inhibitor would increase the sensitivity of cancer cells to paclitaxel. To test this hypothesis, we used flow cytometry to detect the rate of apoptosis. As shown in Fig. 4h, i, treatment of A549T cells with 200 nM paclitaxel for 48 h induced ~10% cell apoptosis. However, when the cells were treated with paclitaxel in combination with AMO-miR-421, there was ~20% cell apoptosis, and the rate of apoptosis was dramatically increased. We next assessed the in vivo efficacy of paclitaxel and AMO-miR-421. A549T cells were injected into nude mice, which were treated daily with paclitaxel for 21 days. As Fig. 4j shows, paclitaxel in combination with AMO-miR-421 exhibited a stronger tumour growth suppression effect than paclitaxel treatment alone. In addition, the mice (*n* = 5) had no significant body weight loss or apparent toxicity after treatment with both paclitaxel and AMO-miR-421, thus excluding the possible toxicity of combination treatment in mice (Fig. 4k). All of these data showed that miR-421 was involved in chemosensitivity in lung cancer, and miR-421 knockdown with an AMO provides a therapeutic intervention strategy to reverse paclitaxel resistance in lung cancer.

### MiR-421 expression is upregulated by β-catenin

**Fig. 5:**
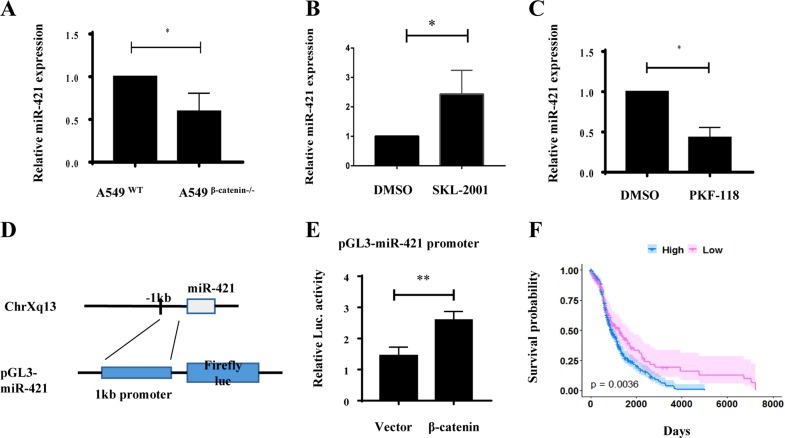
β-catenin upregulates miR-421 expression. **a** Detection of endogenous miR-421 expression in WT or β-catenin knockout A549 cells by real-time PCR. The data are normalized to U6. **b** Examination of endogenous miR-421 expression in A549 cells after treatment with the Wnt agonist SKL-2001 by real-time PCR. **c** Quantification of endogenous miR-421 expression in A549 cells after treatment with the Wnt inhibitor PKF118 by real-time PCR. **d** Chromosomal location of miR-421 on chromosome Xq13. The promoter region (1 kb) was cloned into the luciferase construct pGL3-basic. **e** The control vector or β-catenin and the constructed luciferase vector were co-transferred into HEK293 cells. Firefly luciferase activity was measured 24 h after incubation and normalized to Renilla luciferase activity. The data are expressed as the means ± SD (*n* = 3, *P < 0.05; **P < 0.01) **f** Kaplan–Meier survival analysis for lung cancer patients based on different β-catenin expression levels

The Wnt/β-catenin/TCF4 pathway plays critical roles in cancer progression. β-catenin promotes downstream gene expression by binding TCF to the promoter region of the gene^48,51^. The site recognized by β-catenin/TCF4 contains CTTTG or CTTTG/CAAAG^56^. Several potential β-catenin/TCF-4 binding sites were found to contain this region. We next examined the transcriptional regulation of miR-421 in lung cancer cell lines. To investigate the relationship between β-catenin and miR-421, we performed CRISPR/Cas9-mediated deletion of β-catenin in A549 cells (Supplementary Fig. S3). We screened monoclonal 2F4 cell lines for β-catenin knockout and verified them by WB and DNA sequencing (Supplementary Figs.S3C, and S3D). Knockout of β-catenin decreased miR-421 expression in A549 cells (Fig. 5a). To further clarify the potential role of the Wnt/β-catenin pathway in regulating the expression of miR-421, we examined the effect of SKL-2001, a chemical agonist of the Wnt/β-catenin signalling pathway. Treatment of A549 cells with SKL 2001 significantly increased the expression of miR-421 (Fig. 5b). In contrast, miR-421 expression was significantly reduced upon treatment with a Wnt inhibitor, PKF-118 (Fig. 5c), suggesting that the Wnt/β-catenin pathway participates in the regulation of miR-421. In contrast, we cloned a 1-kb DNA fragment of the miR-421 promoter region into a firefly luciferase reporter construct and examined the effect of β-catenin on the miR-421 promoter region (Fig. 5d). The overexpression of β-catenin significantly activated the luciferase activity of the miR-421 promoter region in HEK293 cells (Fig. 5e). Notably, TCGA data also demonstrated that compared with the patients with low β-catenin expression levels, the patients with β-catenin overexpression had shorter survival times (Fig. 5f). Taken together, these results strongly suggest that β-catenin stimulates miR-421 expression, which in turn downregulates KEAP1 expression in lung cancer.

## Discussion

Lung cancer has the highest morbidity and mortality rates of cancers worldwide. Chemotherapy is the common treatment for lung cancer; however, chemoresistance creates difficulties for its use. In this study, we demonstrated for the first time that (I) miR-421 downregulates KEAP1 expression by targeting the KEAP1 3′UTR; (II) reduced KEAP1 confers drug resistance in NSCLC at least partly via miR-421 overexpression; and (III) miR-421 expression is upregulated by the Wnt/β-catenin signalling pathway.

The key finding of our study is that miR-421 is involved in the modulation of KEAP1 and the downregulation of KEAP1 expression by directly targeting the KEAP1 3′UTR in lung cancer. In previous studies, loss of KEAP1 function promoted tumour growth, suggesting that KEAP1 is a tumour suppressor gene^57,58^. In line with this result, immunohistochemical staining for KEAP1 in 129 lung cancer patient samples showed that the expression level of KEAP1 is related to the clinical stage of the cancer, which also indicates the critical role of KEAP1 in lung tumours (Fig. 2c). We also tried to study the relationship between KEAP1 and patient survival. However, due to the loss of contact with some patients, the number of cases was insufficient, and there were no statistically significant results. However, a trend was observed that low KEAP1 expression is associated with poor survival in squamous carcinoma patients, and squamous carcinoma patients are the subgroups that will use chemotherapy in the clinic. (Supplementary Fig. S4). Moreover, miR-421 expression is upregulated in many types of lung cancer cells (Fig. 2a), suggesting that it is an onco-microRNA in NSCLC. In addition, we demonstrated that overexpression of miR-421 promotes cell migration and tumour growth in vitro and in vivo (Fig. 3). To verify that miR-421 mediates the function of KEAP1, we used a cell line overexpressing KEAP1 for rescue experiments (Supplementary Fig. S2). We also have performed rescue assay in A549 and H1975 cell line, respectively. As shown in Supplementary Fig. S2A and S2B, KEAP1 rescue the defective migration ability in miR-421-treated cells in both A549 and H1975, validating that KEAP1 mediates the function of miR-421. In fact, a number of potential miRNA biomarkers in cancer have been reported^59^. Early lung cancer marker detection is important for clinical guidance. Studies have also reported that plasma or serum miRNAs are stable and suitable for biomarkers^60^. There is an increasing demand to find biomarkers for liquid biopsies to predict treatment outcomes. Our investigation showed that miRNA-421 can potentially be detected in the plasma (Fig. 2B). It may be secreted from tumours by exosomes^61,62^, which may increase its potential for detection. More patient samples should be included for large-scale analysis in future studies. Taken together, our results suggest that miR-421 plays an important role in the progression of lung cancer and that miR-421 is a potential biomarker for lung cancer.

Our study further showed that KEAP1-mediated resistance to paclitaxel is regulated by miR-421. Previous studies have shown that reducing the expression of KEAP1 reduces sensitivity to chemotherapy^31,63,64^. However, few studies have reported how KEAP1 is regulated in this process. Our study combines miRNA regulation of KEAP1 with cancer resistance. Our data demonstrate that paclitaxel resistance is related to low KEAP1 expression in lung cancer. Our results also demonstrated that miR-421 is highly expressed in paclitaxel-resistant cells (Fig. 4c). Interestingly, when we used synthetic AMOs o reduce the expression of endogenous miR-421, we detected that A549T cells became paclitaxel-sensitive. Similarly, AMO-421 can also increase the sensitivity of paclitaxel in vivo (Fig. 4). Therefore, targeting miR-421/KEAP1 could be a potential strategy for the treatment of paclitaxel-resistant lung cancer patients.

Another important finding is that we identified that miR-421 expression is upregulated by the Wnt/β-catenin signalling pathway. We found that miR-421 is significantly downregulated in A549 cells when β-catenin is knocked out, which suggests that miR-421 expression is regulated by β-catenin. Previous studies have reported that miRNA-18a and miRNA-421 are positively controlled by mTOR signalling^65^. It has also been reported that N-Myc-induced miR-421 downregulates the expression of ATM (Ataxia telangiectasia-mutated gene), establishing an N-Myc/miR-421/ATM pathway that participates in N-Myc-induced carcinogenesis in neuroblastoma^66^. Given that the Wnt signalling pathway also regulates N-myc, it is possible that β-catenin indirectly regulates miR-421 expression. In addition, we have shown that β-catenin can directly regulate miR-421 expression via luciferase assays (Fig. 5e). In summary, our data elucidated that miR-421 is upregulated directly by β-catenin-mediated transcription.

**Fig. 6:**
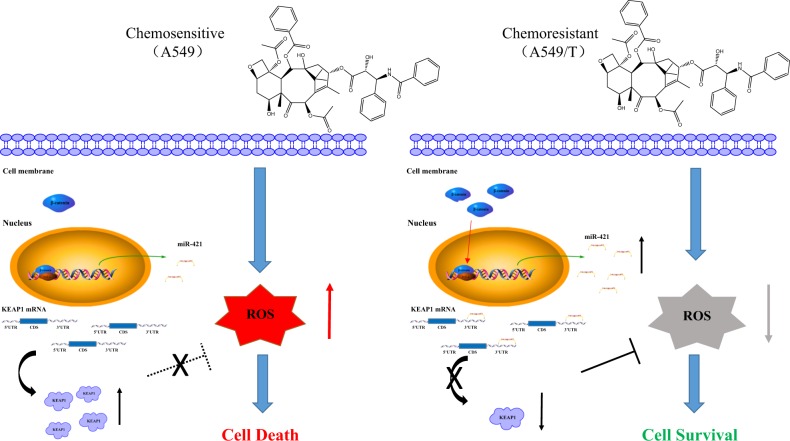
Schematic showing that the β-catenin-miR-421-KEAP1 axis mediates drug resistance in lung cancer

In Fig. 6, we found that miR-421 is overexpressed in lung cancer and focused on the regulation upstream and downstream of miR-421. Crosstalk among various signal pathways is also very meaningful, and several of them are connected by microRNA. Next, we will thoroughly investigate the interactions of the two signalling pathways and the mechanisms of tumour resistance. Notably, a novel β-catenin/miR-421/KEAP1 signalling pathway was further characterized to reveal how the Wnt/β-catenin signalling pathway regulates the antioxidant signalling pathway in drug resistance.

Overall, this study suggested a new role for miRNA-421 and the therapeutic potential for applying AMO-421 for paclitaxel treatment sensitization in NSCLC; nanotags and new formulations of AMO-421, such as ones with exosome binding, are being developed for stabilization of the RNA drug. Recently, small RNA intervention has emerged as new anti-cancer and drug development strategies. Recently, Onpattro (patisiran) was approved by the FDA for the treatment of peripheral nerve disease^67^. The first RNA drug has been approved for the market, but stabilization and delivery to tumour tissues are concerning issues. Our drug is applied intratumourally and demonstrated remarkable tumour suppression; thus, it can potentially be developed into new small RNA targeting drugs for lung cancer in the future.

## Supplementary information

Supplemental Figure 1

Supplemental Figure 2

Supplemental Figure 3

Supplemental Figure 4

Supplementary figures legends
