# Supplementary figures and images for: MicroRNA-421 confers paclitaxel resistance by binding to the KEAP1 3′UTR and predicts poor survival in non-small cell lung cancer
Source: Cell Death Dis. 2019 Oct 28;10(11):821. doi: 10.1038/s41419-019-2031-1 (PMC6817891; doi:10.1038/s41419-019-2031-1)

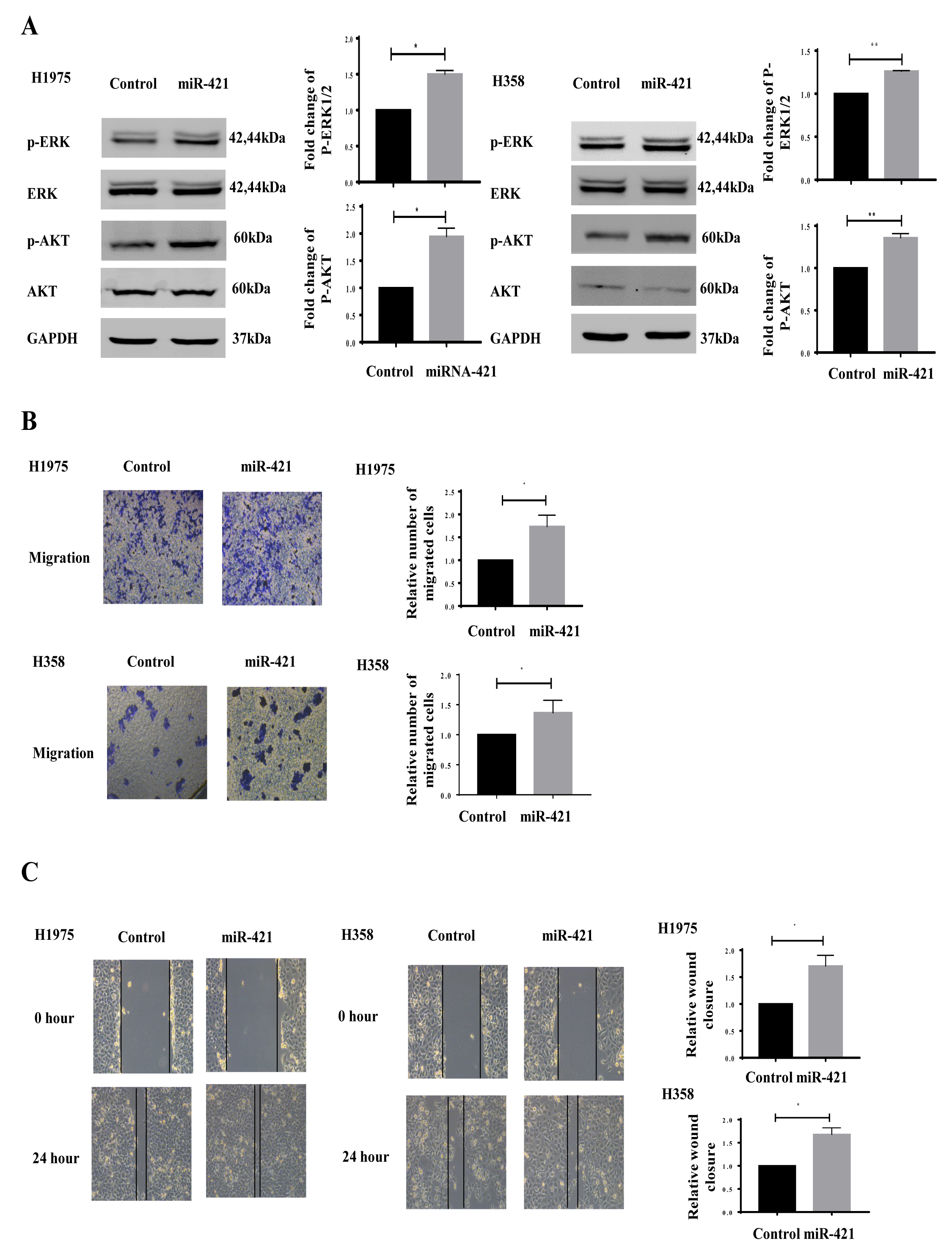

Supplement: Supplementary file 1 — Supplemental Figure 1 [file 41419_2019_2031_MOESM1_ESM.tif]

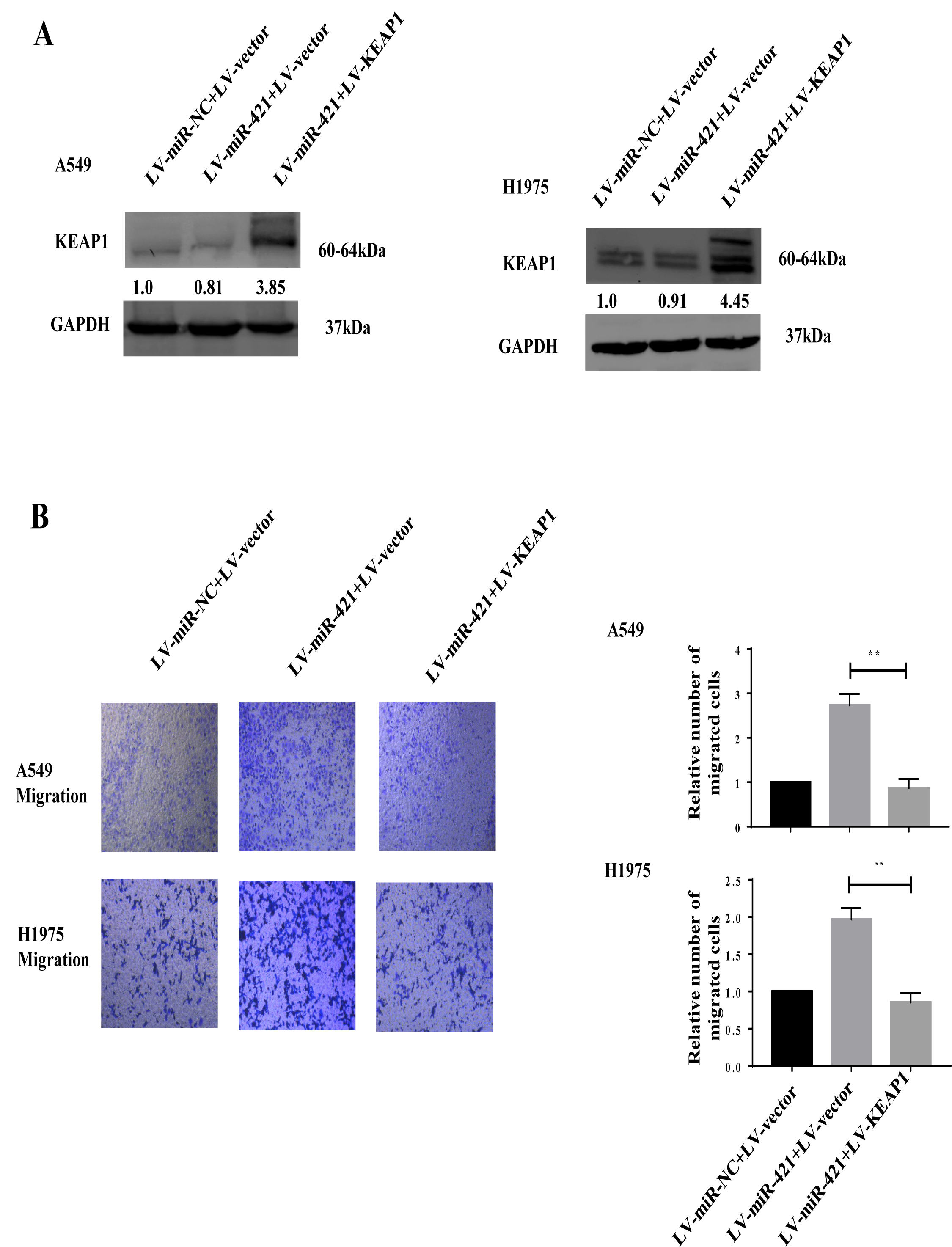

Supplement: Supplementary file 2 — Supplemental Figure 2 [file 41419_2019_2031_MOESM2_ESM.tif]

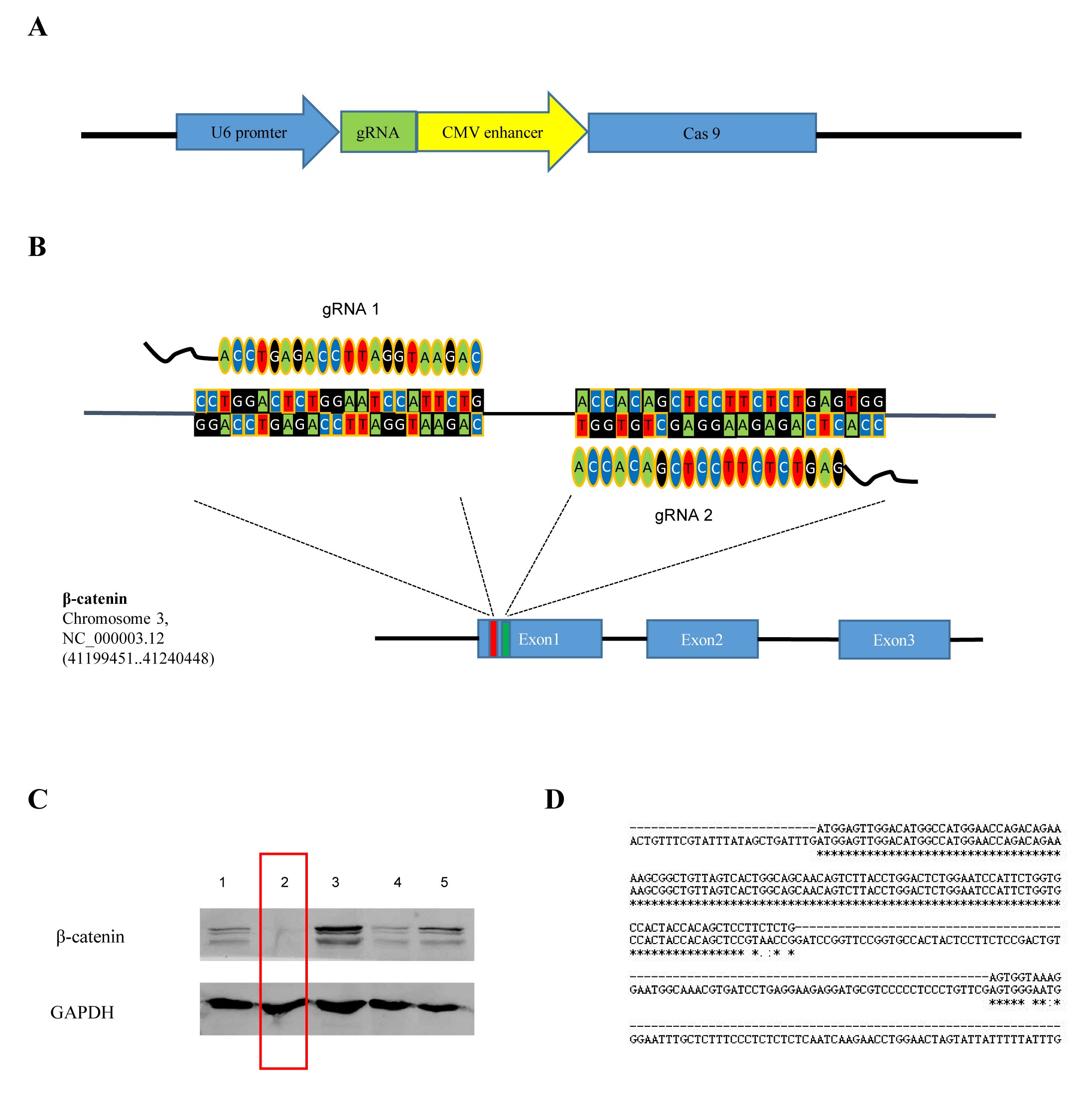

Supplement: Supplementary file 3 — Supplemental Figure 3 [file 41419_2019_2031_MOESM3_ESM.jpg]

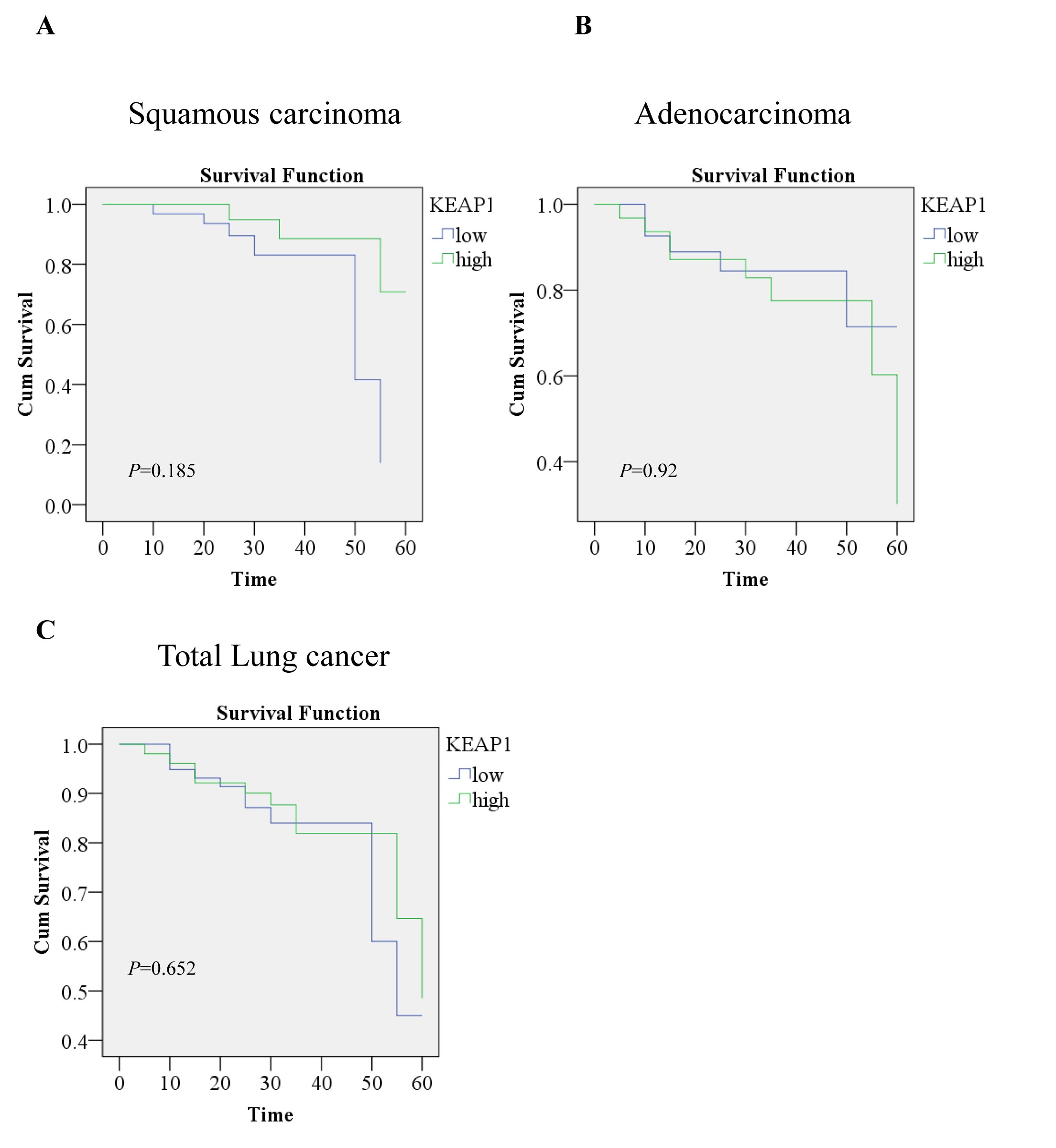

Supplement: Supplementary file 4 — Supplemental Figure 4 [file 41419_2019_2031_MOESM4_ESM.jpg]
