# Supplementary material for: MicroRNA-421 confers paclitaxel resistance by binding to the KEAP1 3′UTR and predicts poor survival in non-small cell lung cancer
Source: Cell Death Dis. 2019 Oct 28;10(11):821. doi: 10.1038/s41419-019-2031-1 (PMC6817891; doi:10.1038/s41419-019-2031-1)
Supplement: Supplementary file 5 — Supplementary figures legends [file 41419_2019_2031_MOESM5_ESM.docx]

**Figure S1. MiR-421 plays an oncogenic role in NSCLC.** (A) MiR-421 increases the level of p-AKT and p-ERK in H1975 (left panel) and H358 (right panel) cell line. The cell extracts were prepared and analyzed by western blotting with antibodies against p-ERK, p-AKT, ERK, AKT. GAPDH was used as a loading control. (B) Migration assay was performed in Negative control and miR-421 overexpression H1975 cell (upper panel) and H358 cells (lower panel), respectively. Data are presented as mean ± SD. (C) Wound healing assay in H1975 cell (left panel) and H358 cells (right panel) with Negative control or miR-421 overexpression. Images were taken 0 h and 24 h respectively after wounded. Data are presented as mean ± SD.

**Figure S2. KEAP1 reduces cell migrate in miR-421 treated cells.** (A) Western blotting analysis verifying the protein level of KEAP1. (B) Transwell assay were performed in both A549 and H1975. Data are presented as mean ± SD.

**Figure S3. Knockout of β-catenin in A549 cell lines using the CRISPR/Cas9 system.** (A) Design of sgRNAs for β-catenin. Two sgRNAs were designed for β-catenin. Sequencing data showed that these sgRNAs were appropriately inserted into the pX335-U6-Chimeric_ BB-CBh-hSpCas9n (D10A) vector. (B) WB data showing that monoclonal 2F4 cells were significantly depleted in the A549 cell lines. (C) Sequencing data showing that the monoclonal 2F4 cell genome was mutated.

**Figure S4. Relationship between KEAP1 and non-small cell lung cancer survival rates.**
